# Association of executive function with suicidality based on resting-state functional connectivity in young adults with subthreshold depression

**DOI:** 10.1038/s41598-023-48160-y

**Published:** 2023-11-24

**Authors:** Je-Yeon Yun, Soo-Hee Choi, Susan Park, Joon Hwan Jang

**Affiliations:** 1 Seoul National University Hospital, Seoul, Republic of Korea; 2 Yeongeon Student Support Center, Seoul National University College of Medicine, Seoul, Republic of Korea; 3 Department of Psychiatry, Seoul National University Hospital, Seoul, Republic of Korea; 4 Department of Psychiatry, Seoul National University College of Medicine, Seoul, Republic of Korea; 5 Department of Psychiatry, Seoul National University Health Service Center, 1 Gwanak-Ro, Gwanak-Gu, 08826 Seoul, Republic of Korea; 6 Department of Human Systems Medicine, Seoul National University College of Medicine, Seoul, Republic of Korea

**Keywords:** Depression, Human behaviour, Diagnostic markers, Depression, Brain imaging, Cognitive control, Problem solving, Network models

## Abstract

Subthreshold depression (StD) is associated an increased risk of developing major depressive disorder (MDD) and suicidality. Suicidality could be linked to distress intolerance and use of context-dependent strategies. We identified neural correlates of executive functioning among the hubs in the resting-state functional connectome (rs-FCN) and examined associations with recent suicidality in StD and MDD. In total, 79 young adults [27 StD, 30 MDD, and 23 healthy controls (HC)] were scanned using magnetic resonance imaging. Neurocognitive measures of the mean latency to correct five moves in the One Touch Stockings of Cambridge (OTSMLC5), spatial working memory between errors (SWMBE), rapid visual information processing A′ (RVPA′), and the stop signal reaction time in the stop signal test (SSTSSRT) were obtained. Global graph metrics were calculated to measure the network integration, segregation, and their balance in the rs-FCN. Regional graph metrics reflecting the number of neighbors (degree centrality; DC), participation in the shortcuts (betweenness centrality; BC), and accessibility to intersections (eigenvector centrality; EC) in the rs-FCN defined group-level hubs for StD, HC, and MDD, separately. Global network metrics were comparable among the groups (all *P* > 0.05). Among the group-level hubs, regional graph metrics of left dorsal anterior insula (dAI), right dorsomedial prefrontal cortex (dmPFC), right rostral temporal thalamus, right precuneus, and left postcentral/middle temporal/anterior subgenual cingulate cortices were different among the groups. Further, significant associations with neurocognitive measures were found in the right dmPFC with SWMBE, and left dAI with SSTSSRT and RVPA′. Shorter OTSMLC5 was related to the lower centralities of right thalamus and suffer of recent 1-year suicidal ideation (all Ps < 0.05 in ≥ 2 centralities out of DC, BC, and EC). Collectively, salience and thalamic networks underlie spatial strategy and planning, response inhibition, and suicidality in StD and MDD. Anti-suicidal therapies targeting executive function and modulation of salience-thalamic network in StD and MDD are required.

## Introduction

Subthreshold depression (StD) is defined as a clinical syndrome characterized by the presence of two or more depressive symptoms that have been consistently present for at least 2 weeks in individuals who do not meet the criteria for diagnosis of minor depression, major depressive disorder (MDD), or dysthymia^1^. StD has a lifetime prevalence as high as 26%^2^. Likewise in MDD, individuals with StD also show impairment in social functioning^3^ and suffer suicidal ideation^4^. Therefore, individuals with StD require health care utilization similar to that required by MDD^5^. Regarding the structural and functional neural foundation of StD compared to HC and MDD, first, gray matter morphology and/or cortical thickness of the superior-middle temporal gyri, hippocampus, amygdala, anterior cingulate gyrus, medial orbitofrontal and superior frontal gyri, and cuneus could differentiate StD in their mid-teens from both HC and MDD^6^. Second, StD in their early 20 s demonstrate significantly reduced microstructural integrity in specific white matter tracts including the pontine crossing tract, genu of the corpus callosum, posterior limb of the internal capsule, and anterior/posterior/superior corona radiata compared with HC^7^. Notably, the resting-state functional connectivity (rs-FC) between the left amygdala and the middle frontal gyri and insula^8^, and between the dorsolateral prefrontal cortex and the temporoparietal junction, precuneus, and anterior insula, is weaker in StD in their early 20 s to early 30 s than in HC^9^. Conversely, stronger rs-FC of StD is found between the left amygdala and left precuneus^8^, and between the default mode network and the ventral striatum^10^, than in HC. Moreover, the degree of depressive symptom reduction in patients in their early 20 s with StD after attentional bias modification training was proportional to reduced rs-FC between the right anterior insula and right frontoinsular and supramarginal cortices^11^. In another study, enhanced health-related quality of life in patients in their late teens with StD after behavioral activation sessions showed a negative correlation with reduced rs-FC between the anterior default mode subnetwork and the dorsal anterior cingulate cortex^12^. In short, structural–functional characteristics of brain network could not only distinguish StD from HC and MDD but also underlie the treatment-related improvement of depressive symptoms in StD.

Although more severe depressive symptoms exhibit increased concurrent suicidal ideation^13,14^, StD and anxiety are also associated with an increased prevalence of current suicidal ideation^14,15^ and lifetime suicidal attempts^4^. First, suicide attempts are positively and significantly associated with a history of suicide planning and/or intention to act, difficulty controlling suicidal thoughts during the worst week of ideation, and non-suicidal self-injury^16^. Second, cognitive-affective styles of distress intolerance, heightened emotional reactivity, a propensity for risk-taking in decision-making, and impaired reward/punishment-based learning are associated with an increased risk of suicidal ideation and attempts^17–19^. A negative association between daily ratings of psychological pain and functional activation of the orbitofrontal cortex during social exclusion of euthymic females with a history of suicide attempts and previous major depressive episodes^20^ suggests a possible role of tolerance of emotional distress as a mediator between stressful life events and suicide risk in MDD^21^. MDD who have a history of suicide attempts may struggle to improve their problem-solving strategies. This difficulty could be attributed to their impaired ability in probabilistic reversal learning and their tendency to base decisions on feedback from the most recent trial rather than considering the overall context^19^. Regarding decision-making with conflict control, suicidal ideation in StD is reflected in the functional activation of the thalamus during response generation against a possible larger monetary loss^22^. In addition, a greater overall proportion of betting and risk-taking in decision-making during the Cambridge Gamble Task has been found in suicidal ideation and anxiety and/or mood disorders^18^. Third, characteristics of resilience^23^ such as personal competence, tenacity, tolerance of negative affect, positive acceptance of change, and secure relationships are inversely correlated with depressive symptoms and suicidality^24^. Collectively, associations of suicidal risk with suicidal ideation, cognitive and affective style, and task-related functional brain activation in depression have been reported.

On the contrary, few information is available regarding the neural correlates of associations between suicidal ideation versus executive functioning of distress tolerance (response inhibition and sustained attention) and strategy-based decision-making (visuospatial strategy and planning) not only in StD^5,25^ but also in MDD^3,26,27^. Of note, clinical symptoms of and neurocognitive functioning in psychiatric disorders could be explained by alterations in, or abnormal integration of, spatially distributed brain regions that would normally comprise a large-scale network subserving function^28,29^. Therefore, this study derived global and local graph metrics of graph theory^30^, and firstly aimed to find the group-level hubs having numerous neighbors (degree centrality; DC) and more access to intersections (eigenvector centrality; EC), and frequently participating in the shortcuts (betweenness centrality; BC) in the rs-FCN of StD, MDD, and HC separately. Second, this study aimed to uncover the possible neural correlates of neurocognitive functioning among the group-level hubs with significant between-group differences in 2 ≥ centralities (out of DC, BC, and EC). Third, this study also examined possible associations of executive functioning (visuospatial working memory and strategy, spatial planning, sustained attention, and response inhibition) with recent 1-year suicidal ideation.

We firstly hypothesized that the rs-FCN of StD would show centrality different from that of HC and MDD in hub regions comprising the dorsal attention network^31,32^, ventral attention or salience network^33^, default mode network^34,35^, thalamus^36^, and somatomotor network^32,37^. Second, among these hubs, we expected to find neural correlates of executive function in rs-FCN regions of the prefrontal, anterior, and posterior cingulate, insula, and thalamus of StD, HC, and MDD^38,39^. Third, considering the associations among executive dysfunction, hopelessness, and suicidality^40,41^, we also expected to elucidate the association between recent (past year) suicidal ideation versus executive function in StD and MDD. In regards of the possible staging concept of psychiatric disorders including mood disorder^42^, we hypothesized that neural underpinning of suicidality-executive functioning association would be shared between the StD and MDD.

## Methods

### Participants and clinical measurements

A total of 79 undergraduate or graduate students (HC, n = 23; MDD, n = 30; StD, n = 26; Table 1) participated in this study between October 2017 and September 2020 at Seoul National University (SNU), Seoul, Republic of Korea. For recruiting the study participants among the total student population of N = 42,603 (https://diversity.snu.ac.kr/page/dashboard.php?lang=en), promotional flyers were distributed using the mass e-mails through the portal system of SNU, were displayed on campus bulletin boards, and were provided to participants of annual health examination program and public lectures on campus. All participants provided written informed consent prior to enrollment. Participants’ anonymity has been preserved. All participants satisfied the following inclusion criteria: (1) 18–35 years of age; (2) no lifetime diagnosis of psychotic disorder, substance use disorder, or loss of consciousness due to head injury; and (3) no use of psychotropic medication within 8 weeks before study participation. Diagnosis of psychiatric disorders (either MDD or StD) or exclusion of lifetime history or current morbidity of psychiatric disorders (for HC) were made based on semi-structured interviews using the MINI-International Neuropsychiatric Interview^43,44^ and clinical decision by licensed psychiatrists. At the time of study participation, current experience of a depressive mood and/or loss of interest or pleasure during the previous 2 weeks was reported by all MDD (n = 30) and StD (n = 26). Further, they also satisfied five or more components (MDD) or two to four components (StD) of item A of the diagnostic criteria for MDD in the fifth edition of the Diagnostic and Statistical Manual of Mental Disorders^45^. For all participants of StD, MDD, and HC, self-reporting measures of the 9-question Patient Health Questionnaire (PHQ-9)^46,47^ and the 7-item General Anxiety Disorder questionnaire (GAD-7)^48,49^ were applied to measure the depressive symptoms [sadness/hopelessness, anhedonia, insomnia, appetite change, psychomotor changes, fatigue, self-reproach, concentration difficulty, suicidality (thoughts of self-harm or suicide)] and anxiety, respectively. The internal consistency measured by way of the Cronbach’s alpha were 0.95 for the PHQ-9 and 0.88 for the GAD-7^47,49^. This study was approved by the Institutional Review Board of Seoul National University College of Medicine and Hospital (Seoul, Republic of Korea; No. 1608–079-785) and was performed in accordance with the ethical standards of the 1975 Declaration of Helsinki and its later amendments in 2013.

**Table 1:** Demographics, clinical, and neurocognitive characteristics.

	Healthy control (HC; N = 23)		Subthreshold depression (StD; N = 26)		Major depressive disorder (MDD; N = 30)		Group effects		Post-hoc pairwise comparisons (*P* values)		
	Mean	SD	Mean	SD	Mean	SD	F/chi^2	*P* value	HC versus StD	HC versus MDD	StD versus MDD
Age	24.7	2.9	24.2	3.6	24.4	3.1	0.129	0.880	0.870	0.944	0.977
Sex (M/F)	13/10		14/12		15/15		0.230	0.892	0.000	0.000	0.000
Education	16.7	2.1	16.5	2.2	16.9	2.5	0.222	0.801	0.954	0.938	0.785
PHQ-9 total score	3.2	2.6	8.0	3.6	11.9	4.2	38.029	< 0.001	0.000	0.000	0.000
GAD-7 total score	2.3	2.3	5.2	3.9	8.6	4.7	17.512	< 0.001	0.030	0.000	0.004
OTSMLC5	37,403.1	24,962.0	27,529.5	18,098.0	27,969.4	18,904.3	1.787	0.174	0.222	0.230	0.997
SWMBE	12.0	13.1	8.2	9.3	10.4	9.9	0.767	0.468	0.442	0.859	0.725
SSTSSRT	193.2	22.8	211.5	26.9	198.4	30.2	3.035	0.054	0.054	0.771	0.175
RVPA’	1.0	0.0	1.0	0.0	1.0	0.0	0.741	0.480	0.631	0.473	0.971
Recent 1-year suicidal ideation (N/Y)	23/0		14/12		16/14		15.919	< 0.001	< 0.001	< 0.001	0.969

PHQ-9, Patient Health Questionnaire-9; GAD-7, 7-item General Anxiety Disorder questionnaire; OTSMLC5, mean latency to correct five moves in the One Touch Stockings of Cambridge; SWMBE, spatial working memory between errors; RVPA’, rapid visual information processing A′; SSTSSRT, stop signal reaction time in the stop signal test.

### Magnetic resonance imaging (MRI)

All participants underwent MRI using a 3-Tesla scanner (MAGNETOM TrioTim syngo MR B17; Siemens Healthineers, Erlangen, Germany) to evaluate the whole-brain anatomy and rs-FC. The whole-brain anatomy was acquired with a high-resolution T1-weighted, three-dimensional magnetization-prepared rapid acquisition gradient echo sequence (repetition time = 1670 ms, echo time = 1.89 ms, field of view = 250 mm, flip angle = 9°, number of slices = 208, and voxel size = 1.0 × 1.0 × 1.0 mm^3^). During the acquisition of resting-state functional MRI data, the participants were asked to relax with their eyes closed but to remain awake (repetition time = 2000 ms, echo time = 30 ms, flip angle = 80°, number of slices = 34, and voxel size = 3.4 × 3.4 × 3.4 mm^3^).

### Neuropsychological tests

Four tasks from the Cambridge Neuropsychological Test Automated Battery (CANTAB) were applied in this study: rapid visual information processing (RVP), spatial working memory (SWM), the One Touch Stockings of Cambridge (OTS) task, and the stop signal test (SST). Among several outcome measures, four (one from each task) were selected as representative of higher neurocognitive function (Table 1): (1) sustained attention [RVPA′ (RVPA′): a measure of sensitivity to a target (regardless of response tendency), calculated as 0.5 + [(h − f) + (h − f)2] / [4 × h × (1 − f)]), where h = probability of hits (correct responses) and f = probability of false alarms (inappropriate responses) divided by the sum of total false alarms and total correct rejections]^50,51^, (2) visuospatial working memory and strategy [SWM between errors (SWMBE): number of times the participant revisited a box where a token had previously been found]^52^, (3) spatial planning [mean latency to make five correct moves in the OTS (OTSMLC5): the mean latency between the appearance of the balls and the choice of the correct box via five moves]^53^, and (4) response inhibition [stop signal reaction time in the SST (SSTSSRT): the delay between the instructions and the stop signal, adjusted for individual performance (i.e., the 50% response inhibition failure rate)^54^.

### Resting-state functional MRI: Preprocessing and construction of the rs-FCN

The first four volumes (corresponding to 8 s) were removed to allow for signal stabilization. Preprocessing of anatomical and functional MRI data was performed using ENIGMA HALFpipe software (version 1.2.1)^55^, which implements fMRIPrep^56,57^. Afterwards, for each participant and time point, average resting-state time series data were extracted from 210 cortical and 36 subcortical brain regions (5-mm-radius spheres) comprising the Human Brainnetome Atlas^58,59^, which is a probabilistic tractography-based finer-grained version of the Desikan–Killiany Atlas^60^. In accordance with consensus partitioning of the rs-FCN reported by Yeo et al^61^. in 2011, each brain region or node was assigned to the seven cortical subnetworks (default-mode, frontoparietal, limbic, ventral attention, dorsal attention, somatomotor, and visual) and one subcortical network. For each participant, the FCN was created by calculating Pearson’s correlation coefficients between the time series of the 226 nodes (20 regions of interest were excluded from among the initial 246 regions of interest because they were not covered by the resting-state functional MRI scan of one or more participants), followed by Fisher’s r-to-z transformation [= $0.5\times [ln\left(1+r\right)-ln\left(1-r\right)]$]^59,62^. Considering their ambiguous interpretation^63^ and detrimental effects on test–retest reliability^64^, the negative-edge weight values (Fisher’s z-transformed correlation coefficients) were set to zero^65,66^.

### Graph theory approach to rs-FCNs: Global network metrics

For examining the global network topology of rs-FCN, the following five global graph metrics of network segregation (γ, Q), integration (λ, GE), and their balance (σ) were calculated using the binary version of rs-FCNs across the network sparsity range of 0.05–0.30 in steps of 0.01 (in connectomes with a sparsity level of 0.05, only the top 5% ranked edges in terms of the edge values survived)^67,68^.

- Normalized clustering coefficient (γ): a measure of the tendency of brain regions within a network to form interconnected groups of three neighboring nodes, normalized by comparing it to the average value of the same measure calculated from 1000 random networks; these random networks are essentially randomized versions of the original network, but they preserve the distribution of connections between nodes^69,70^
- Normalized characteristic path length (λ): average shortest path length between different nodes within a network, normalized by the averaged value of the same variable derived from the 1000 random networks^69,71^
- Small-worldness (σ = γ/λ): a measure of the balance between segregation and integration in the brain network^71^
- Normalized global efficiency (GE): a measure of network integration based on the minimum number of steps separating nodes from each other, normalized by the averaged value of the same variable derived from the 1000 random networks^69,71–73^
- Modularity (Q): the degree to which the network is subdivided into specific modules with denser intra- than inter-modular connections^74–76^

**Figure 1:**
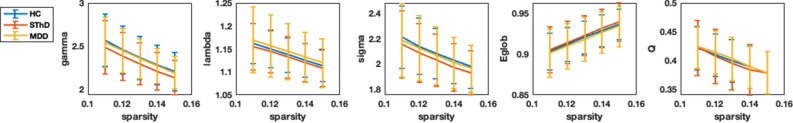
Global graph metrics of the resting-state functional connectome. Five global graph metrics of normalized clustering coefficient (gamma), normalized characteristic path length (lambda), small-worldness (Sigma), normalized global efficiency (GE), and modularity (Q) were calculated. Ranges of network sparsity satisfying the (1) network connectedness (presence of ≥ 1 connections with other brain regions in > 98% of nodes), (2) small-worldness (sigma > 1), and (3) modular organization (Q > 0.3) in all HC (N = 23) were found for the rs-FCN (K = 0.11–0.15). Mean and standard deviation values of each global graph metrics (y-axis) were depicted per group [blue for healthy controls (HC), yellow for major depressive disorder (MDD), and red for sub-threshold depression (StD)] and per network sparsity level (x-axis). The area under the curve (AUC) of each global graph metrics were calculated and compared among the HC, MDD, and StD (statistical threshold of significance *P* < 0.05).

Using the analysis of variance (ANOVA) and independent t-tests, the area under the curve values of these global graph metrics calculated for the subset of network sparsity ranges satisfying the (1) network connectedness [presence of ≥ 1 connections with other brain regions in > 98% of nodes], (2) small-worldness (σ > 1), and (3) modular organization (Q > 0.3) in rs-FCNs of all HC, were compared among the HC, MDD, and StD [Fig. 1**;** statistical threshold of significance *P* < 0.05/5(= number of global graph metrics) = 0.01).

### Graph theory approach to rs-FCNs: Regional graph metrics

For finding principal brain regions in the rs-FCN, the following three regional graph metrics reflecting differential facets of principal nodes such as (1) participation in the shortcuts (BC), (2) connection with intersections (EC), and (3) number of neighbors (DC) were calculated^71,77^.

- Betweenness centrality (BC): fraction of all shortest paths in the network that contain a given node; nodes with high BC values participate in a large number of shortest paths
- Eigenvector centrality (EC): self-referential measure of centrality that reflects the presence of connectedness between a given node and other nodes with high EC values
- Degree centrality (DC): the number of connections or edges incident upon a node)

**Figure 2:**
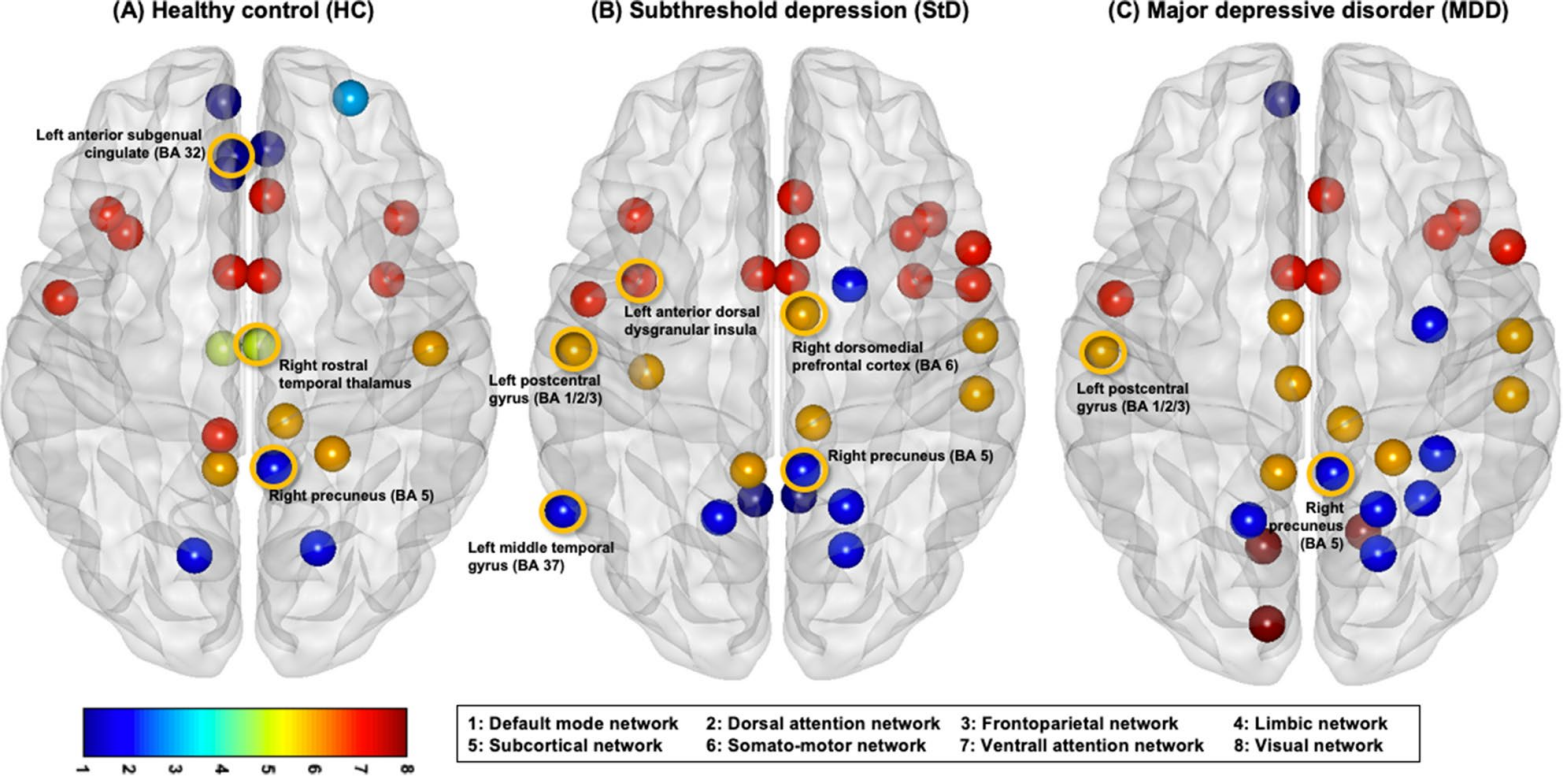
Group-level hubs of resting-state functional connectome (rs-FCN). Group-level hubs most frequently ranked as top 12% in each group of (**A**) HC, (**B**) StD, or (**C**) MDD in 2–3 centralities [among the degree, betweenness, and eigenvector; in the network sparsity range of K = 0.11–0.15 that satisfied three criteria of network connectedness, small-worldness, and modular organization in the rs-FCN of all HC (n = 23)] are displayed. Nodes (circles) are colored to display the 7 cortical and 1 subcortical sub-networks or modules. Among these 47 group-level hubs (comprised of 23 HC hubs, 27 StD hubs, and 26 MDD hubs), seven nodes that showed significant between-group differences in the z-transformed centralities (*P* < 0.05 in 2–3 centralities) are marked with thicker orange-colored rims. First, right precuneus (BA 5) was one of the 9 hubs shared by all three groups; compared to MDD, z-transformed of centralities were higher in StD. Second, among the 7 hubs shared in both MDD and StD, centralities of left postcentral gyrus (BA 1/2/3) were higher in MDD than StD and HC. Third, among the 9 regions ranked as hubs only in StD, centrality values of left anterior dorsal dysgranular insula and right dorsomedial prefrontal cortex (BA 6) were higher in StD compared to both MDD and HC. In addition, centralities of left middle temporal gyrus (BA 37) was higher in StD compared to MDD. Fourth, among the 12 regions ranked as hubs only in HC, centralities of right rostral temporal thalamus were lower in MDD than HC; also, centrality values of left anterior sub-genual cingulate (BA 32) was higher in HC compared to both MDD and StD.

Because the distribution of centrality values is not normal in a scale-free network, these regional graph metrics were z-score-normalized (using the mean and standard deviation of each centralities for HC, MDD, and StD separately) at network sparsity ranges satisfying the network connectedness, small-worldness, and modular organization in rs-FCN of all HC (refer to the ‘Graph theory approach to rs-FCNs: Global network metrics’ in the ‘Methods’), and then averaged for each participant to derive the participant-level centrality values^68^. Brain regions ranked in the top 12% (= 226 nodes × 0.12 ≈ 26–27 nodes) for two or three centralities (out of BC, EC, and DC) in terms of the group-averaged participant-level centrality values were defined as group-level hubs^68,78^ for HC, MDD, and StD separately (Fig. 2). Between-group comparisons of z-transformed centralities (BC, EC, and DC) using the ANOVA and post-hoc independent t-tests were performed only for these group-level hubs; those with significantly different z-transformed centralities (*P* < 0.05) for ≥ 2 centralities (out of BC, EC, and DC) were defined as brain regions having significant between-group differences in hubness (Table 2 and Fig. 2).

**Table 2:** Between-group comparisons of hubs.

Brain region	Laterality	BA	No. of top 12%-ranked centralities (among the EC, BD, and DC)			Mean of eigenvector centrality (EC)			Mean of betweenness centrality (BC)			Mean of degree centrality (DC)			Group effects (*P* values)			Post-hoc pairwise comparisons									
																		HC versus StD (*P* values)			HC versus MDD (*P* values)			StD versus MDD (*P* values)			Between-group difference of Hubness
			HC	StD	MDD	HC	StD	MDD	HC	StD	MDD	HC	StD	MDD	EC	BC	DC	EC	BC	DC	EC	BC	DC	EC	BC	DC	
Precuneus	R	5	**3**^**a**^	**3**^**a**^	**2**^**a**^	0.67	1.28	0.63	0.81	1.15	0.52	0.80	1.21	0.44	**0.033**^**b**^	0.123	**0.008**^**b**^	**0.038**^**b**^	NA	0.110	0.892	NA	NA	**0.023**^**b**^	NA	**0.003**^**b**^	StD > HC = MDD
Anterior dorsal dysgranular insula	L	–	1	**3**^**a**^	1	0.71	1.48	0.61	0.17	0.80	0.29	0.45	1.27	0.50	**0.007**^**b**^	0.063	**0.007**^**b**^	**0.011**^**b**^	NA	**0.005**^**b**^	0.739	NA	NA	**0.005**^**b**^	NA	**0.008**^**b**^	StD > HC = MDD
Dorsomedial prefrontal cortex	R	6	1	**3**^**a**^	0	0.53	1.22	0.48	0.32	0.50	0.18	0.30	0.94	0.31	**0.022**^**b**^	0.450	**0.047**^**b**^	**0.031**^**b**^	NA	**0.043**^**b**^	0.864	NA	NA	**0.010**^**b**^	NA	**0.020**^**b**^	StD > HC = MDD
Middle Temporal Gyrus	L	37	0	**3**^**a**^	0	0.31	0.89	0.08	0.03	0.61	− 0.04	0.20	0.80	− 0.06	**0.028**^**b**^	0.056	**0.010**^**b**^	0.116	NA	0.080	0.417	NA	NA	**0.012**^**b**^	NA	**0.005**^**b**^	StD > HC = MDD
Postcentral Gyrus	L	1/2/3	1	**2**^**a**^	**3**^**a**^	0.47	0.92	1.13	0.55	0.28	1.13	0.44	0.75	1.23	0.073	**0.007**^**b**^	**0.014**^**b**^	NA	0.318	0.275	NA	0.056	0.056	NA	**0.002**^**b**^	0.070	NS
Anterior sub-genual cingulate	L	32	**2**^**a**^	0	0	0.17	− 0.49	− 0.31	0.58	− 0.03	0.23	0.67	0.05	0.21	**0.003**^**b**^	**0.017**^**b**^	**0.006**^**b**^	**0.005**^**b**^	**0.010**^**b**^	**0.005**^**b**^	**0.040**^**b**^	0.116	0.116	0.204	0.146	0.346	MDD = StD < HC
Rostral temporal thalamus	R	–	**2**^**a**^	0	0	0.66	0.05	− 0.23	0.12	0.34	− 0.31	0.59	0.08	− 0.23	**0.009**^**b**^	**0.049**^**b**^	**0.010**^**b**^	0.064	0.503	0.079	**0.006**^**b**^	0.061	0.061	0.260	**0.029**^**b**^	0.204	NS

Group-level hubs that showed significant between-group differences of z-transformed centralities (degree, betweenness, and eigenvector) averaged in the network sparsity levels of K = 0.11–0.15 [that satisfied network connectedness, small-worldness, and modular organization in the resting-state functional connectivity network (rs-FCN) of all healthy controls (HC; N = 23)]. Only 47 brain regions (out of the 226 brain regions comprising the rs-FCN) ranked as group-level hubs either in HC (23 hubs), subthreshold depression (StD; N = 26, 27 hubs) or major depressive disorder (MDD; N = 30, 26 hubs) hubs underwent between-group comparisons. Threshold of statistical significance was set as *P* < 0.05 in 2–3 centralities.

L, Left cerebral hemisphere; R, Right cerebral hemisphere; BA, Brodmann area; EC, Eigenvector centrality; BC, Betweenness centrality; DC, Degree centrality; HC, Health control; StD, Subthreshold depression; MDD, Major depressive disorder.

^a^Group-level hubs.

^b^*P* < 0.05.Significant values are in bold.

**Figure 3:**
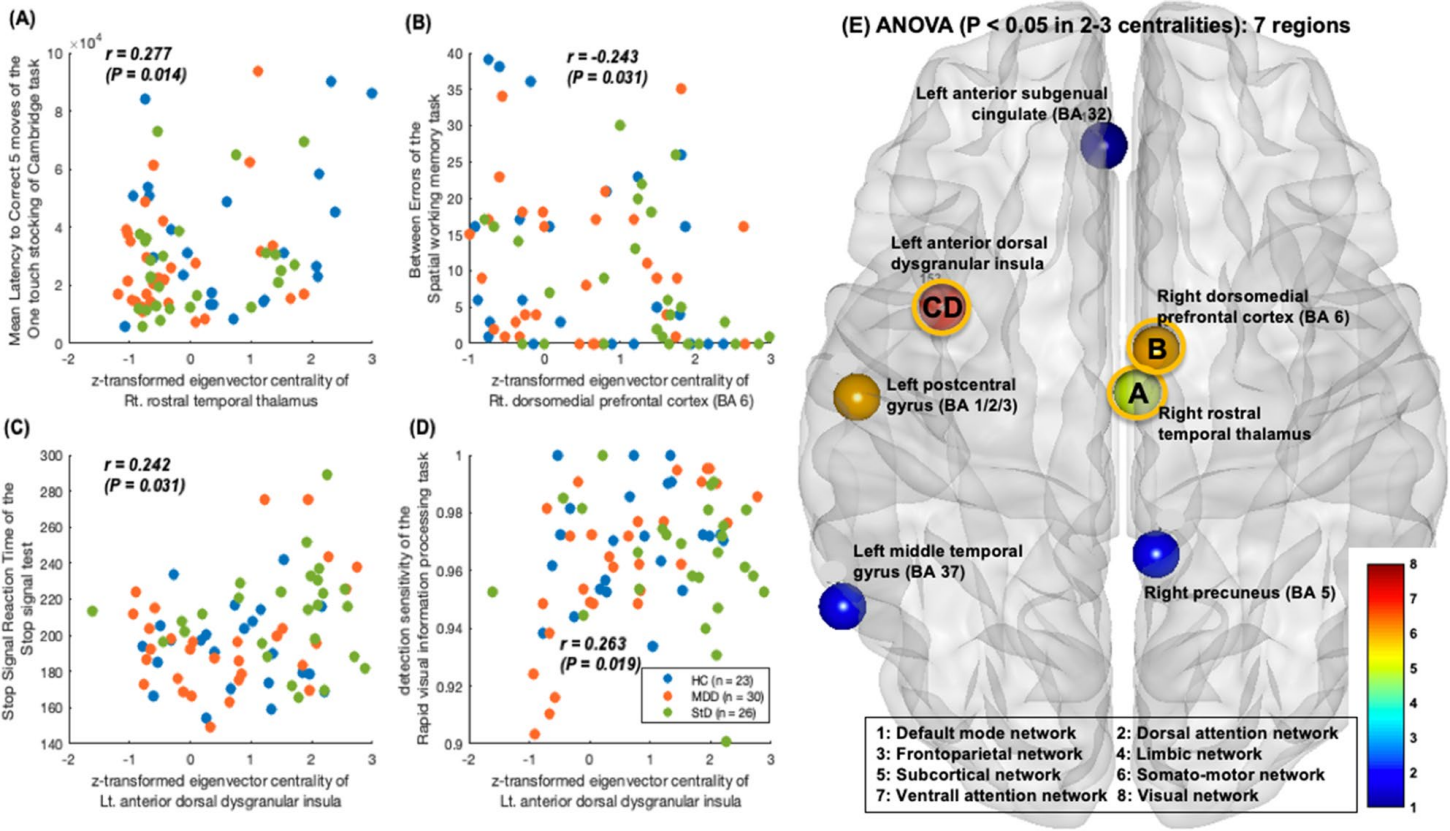
Associations of centralities in hub brain regions with higher neurocognitive performance. (**A**) A positive association was found between the OTSMLC5 (Mean Latency to Correct 5 moves in the One touch stocking of Cambridge task; spatial planning) and z-transformed centralities of right rostral temporal thalamus (r = 0.262 and *P* = 0.020 for degree centrality; r = 0.277 and *P* = 0.014 for eigenvector centrality). (**B**) A negative association between the SWMBE (Between Errors of the Spatial working memory task; visuospatial working memory and strategy) and z-transformed centralities of right dorsomedial prefrontal cortex (BA 6) (r = − 0.317 and *P* = 0.004 for degree centrality; r = − 0.243 and *P* = 0.031 for eigenvector centrality). (**C**) Positive correlation of z-transformed centralities in the left anterior dorsal dysgranular insula with the SSTSSRT (Stop Signal Reaction Time of the Stop signal test; response inhibition; r = 0.228 and *P* = 0.044 for degree centrality, r = 0.242 and *P* = 0.031 for eigenvector centrality). (**D**) Positive correlation of z-transformed centralities in the left anterior dorsal dysgranular insula with the and RVPA (detection sensitivity in the Rapid visual information processing task; sustained attention; r = 0.245 and *P* = 0.030 for degree centrality, r = 0.263 and *P* = 0.019 for eigenvector centrality). **(E)** Seven group-level hubs that showed significant between-group differences of z-transformed centralities (degree, betweenness, and eigenvector) averaged in the network sparsity levels of K = 0.11–0.15 [that satisfied all three criteria of network connectedness, small-worldness, and modular organization in the rs-FCN of all HC individuals (n = 23)]. Only 47 brain regions (out of the 226 brain regions comprising the rs-FCN) ranked as group-level hubs either in HC (23 hubs), StD (27 hubs) or MDD (26 hubs) hubs underwent between-group comparisons. Threshold of statistical significance was set as *P* < 0.05 in 2–3 centralities. Three brain regions that demonstrated significant associations of 2–3 centralities with higher neuropsychological functioning (as shown in **A–D**) are tagged with thicker rims.

Further, to elucidate the graph-based neural foundation of higher neurocognitive functioning in the StD, MDD, and HC groups, Pearson’s correlation coefficients of z-transformed centralities in these group-level hubs having significant between-group differences in hubness with four higher-neurocognitive function measures including RVPA′ (sustained attention)^50,51^, SWMBE (visuospatial working memory and strategy)^52^, OTSMLC5 (spatial planning)^53^, and SSTSSRT (response inhibition) were calculated (Fig. 3; threshold of statistical significance set as *P* < 0.05 for ≥ 2 centralities out of BC, EC, and DC)^54^. All graph theory measures were calculated using the Brain Connectivity Toolbox^71^ and MATLAB R2022a software (https://kr.mathworks.com).

### Statistical analyses

**Figure 4:**
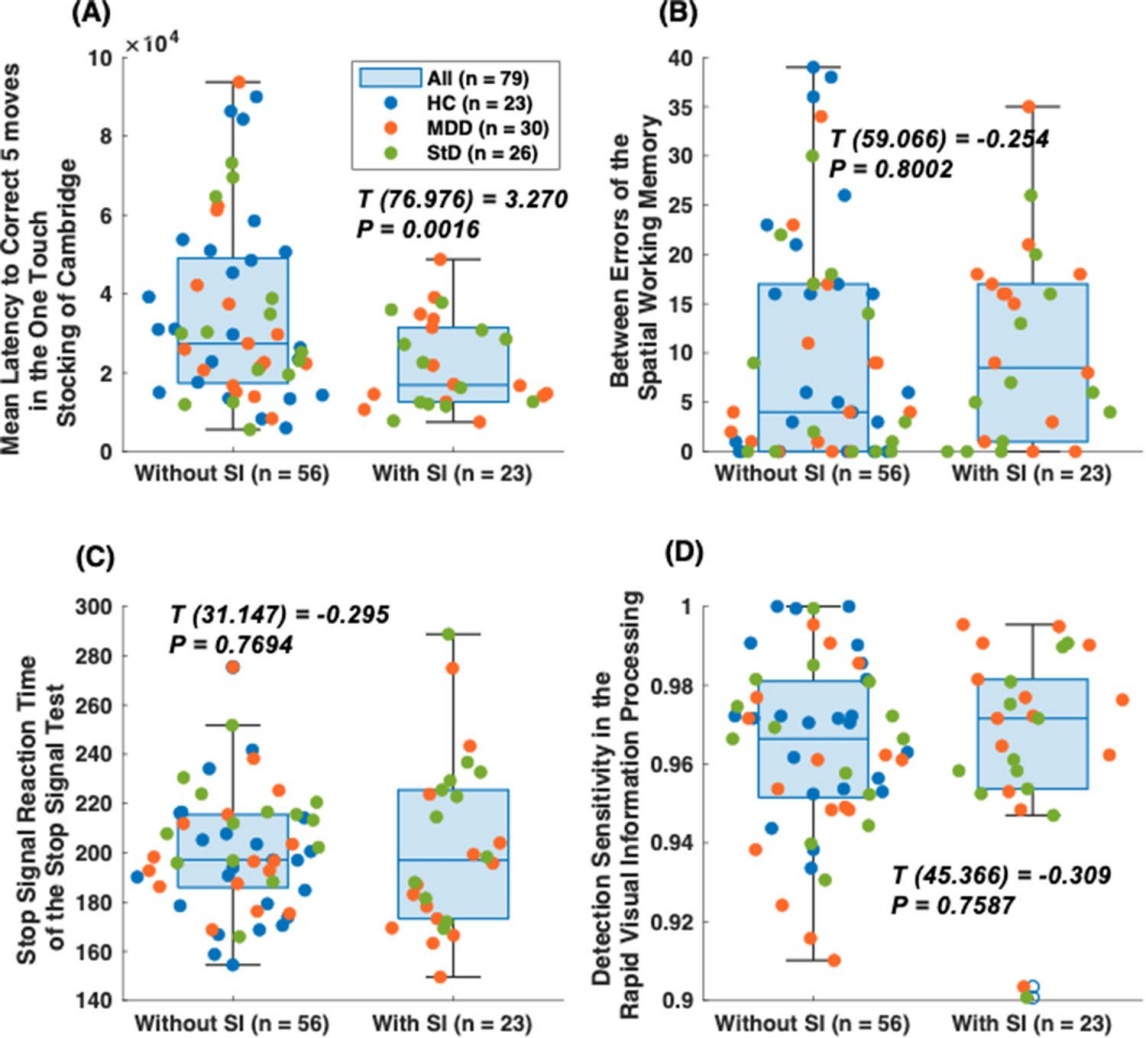
Neurocognitive performance in young adults with [n = 23; subthreshold depression (StD) or major depressive disorder (MDD)] or without [n = 56; StD, MDD, or healthy controls (HC)] suicidal ideation within last year. (**A**) OTSMLC5 (Mean Latency to Correct 5 moves in the One touch stocking of Cambridge task; spatial planning). (**B**) SWMBE (Between Errors of the Spatial working memory task; visuospatial working memory and strategy). (**C**) SSTSSRT (Stop Signal Reaction Time of the Stop signal test; response inhibition). (**D**) RVPA (detection sensitivity in the Rapid visual information processing task; sustained attention).

Between-group comparisons of age, years of education, and total PHQ-9 and GAD-7 scores (Table 1) were performed by way of ANOVA and independent t-tests. The chi-square test was used for between-group comparisons of sex (male/female). The threshold of statistical significance was set at *P* < 0.05. Additionally, neurocognitive measures were compared in terms of the presence or absence of recent 1-year suicidal ideation (Fig. 4). All statistical analyses were performed using MATLAB R2022a software.

## Results

### Participants’ demographic, clinical, and neurocognitive characteristics

Table 1 shows the demographic, clinical, and neurocognitive characteristics of the HC (n = 23), MDD (n = 30), and StD (n = 26). All participants (N = 79) were in their mid-20 s. The study population comprised 42 men and 37 women with an average of 16.7 years of education, and all had remained free of psychotropic medication for > 8 weeks prior to study participation. No statistically significant between-group differences in age, sex, or years of education were found among the HC, MDD, and StD groups (all *P* > 0.05). The four neurocognitive variables (OTSMLC5, SWMBE, SSTSSRT, and RVPA′) showed no significant differences among the three groups (all *P* > 0.05, ANOVA).

However, the severity of depressive and anxiety symptoms differed significantly among the HC, MDD, and StD groups (all *P* < 0.05). Post-hoc analyses also revealed significant between-group differences in depressive symptoms (*P* < 0.001 for HC vs. StD, HC vs. MDD, and StD vs. MDD) and anxiety symptoms (*P* = 0.030 for HC vs. StD, *P* < 0.001 for HC vs. MDD, and *P* = 0.004 for StD vs. MDD). First, MDD experienced moderate depressive symptoms (mean PHQ-9 total score = 12) and mild anxiety symptoms (mean GAD-7 total score = 8.6). Second, StD reported mild depressive symptoms (mean PHQ-9 total score = 7.8) and mild anxiety symptoms (mean GAD-7 total score = 5.2). Third, depressive symptoms (mean PHQ-9 total score = 3.2) and anxiety symptoms (mean GAD-7 total score = 2.3) were mild in HC. Finally, the number of participants with recent (past year) suicidal ideation was zero in the HC group and was similar between the StD and MDD groups (*P* > 0.05).

### Global graph metrics of rs-FCN

All three criteria of network connectedness, small-worldness, and modular organization were satisfied in the network sparsity range of K = 0.11–0.15 for the rs-FCN of all HC. The five global graph metrics [normalized clustering coefficient (γ), normalized characteristic path length (λ), small-worldness (σ = γ/λ), normalized global efficiency (GE), and modularity (Q)] summed in these network sparsity range of K = 0.11–0.15 were comparable among the HC, MDD, and StD groups [F(2) = 0.66 and *P* = 0.521 for γ; F(2) = 0.41 and *P* = 0.664 for λ; F(2) = 0.4 and *P* = 0.672 for σ; F(2) = 0.26 and *P* = 0.769 for GE; and F(2) = 0.15 and *P* = 0.858 for Q] (Fig. 1).

### Regional graph metrics of rs-FCN

Among the 47 group-level hubs (23 HC hubs, 27 StD hubs, and 26 MDD hubs; Fig. 2), 7 nodes showed significant between-group differences in hubness (Table 2). First, the right precuneus [Brodmann area (BA) 5; dorsal attention network] was one of the nine hubs shared by all three groups; the z-transformed values of EC (*P* = 0.024) and DC (*P* = 0.003) were higher in StD than in MDD. Second, among the seven hubs shared by both MDD and StD, the centralities of the left postcentral gyrus (BA 1/2/3; somatomotor network) were higher in MDD than in StD (BC, *P* = 0.002) and HC (DC, *P* = 0.004). Third, among the nine regions ranked as hubs only for StD, the centrality values of the left anterior dorsal dysgranular insula (ventral attention network) [EC, *P* = 0.005 (StD vs. MDD) and *P* = 0.011 (StD vs. HC); DC, *P* = 0.008 (StD vs. MDD) and *P* = 0.005 (StD vs. HC)] and right dorsomedial prefrontal cortex (dmPFC) (BA 6; somatomotor network) [EC, *P* = 0.010 (StD vs. MDD) and *P* = 0.031 (StD vs. HC); DC, *P* = 0.020 (StD vs. MDD) and *P* = 0.043 (StD vs. HC)] were higher in StD than in both MDD and HC. In addition, EC (*P* = 0.012) and DC (*P* = 0.005) of the left middle temporal gyrus (BA 37; dorsal attention network) were higher in StD than in MDD. Fourth, among the 12 regions ranked as hubs only for HC, the centralities of the right rostral temporal thalamus [EC (*P* = 0.006) and DC (*P* = 0.005)] were lower in MDD than in HC. Additionally, the centrality values of the left anterior subgenual cingulate (BA 32; default mode network) were higher in HC than in both MDD [EC (*P* = 0.040) and DC (*P* = 0.022)] and StD [EC (*P* = 0.005), BC (*P* = 0.010), and DC (*P* = 0.005)].

### Associations of hub regions with neuropsychological functioning

Pearson’s correlation coefficients were calculated between the four CANTAB neurocognitive measures (RVPA′, SWMBE, OTSMLC5, and SSTSSRT) and z-transformed centralities in seven group-level hubs with significant between-group differences in hubness (Fig. 3). Notably, a positive association was found between the OTSMLC5 (spatial planning) and z-transformed centralities of the right rostral temporal thalamus [r = 0.262 (*P* = 0.020) for DC, r = 0.277 (*P* = 0.014) for EC]. Moreover, a negative association was found between the SWMBE (visuospatial working memory and strategy) and z-transformed centralities of the right dmPFC (BA 6) [r =  − 0.317 (*P* = 0.004) for DC, r =  − 0.243 (*P* = 0.031) for EC]. Finally, positive correlations were seen between the z-transformed centralities of the left anterior dorsal dysgranular insula and the SSTSSRT (response inhibition) [r = 0.228 (*P* = 0.044) for DC, r = 0.242 (*P* = 0.031) for EC] and RVPA′ (sustained attention) [r = 0.245 (*P* = 0.030) for DC, r = 0.263 (*P* = 0.01) for EC].

Regarding the associations of four executive functioning with suicidal ideation, those with recent 1-year suicidal ideation revealed shorter values of OTSMLC5 [T = 3.270 (*P* = 0.016); Fig. 4A] than those without recent 1-year suicidal ideation. On the contrary, neither the SWMBE [T = − 0.254 (*P* = 0.800); Fig. 4B], the SSTSSRT [T = − 0.295 (*P* = 0.769); Fig. 4C], nor the RVPA′ [T = − 0.309 (*P* = 0.759); Fig. 4D] demonstrated significant differences regarding the presence/absence of recent 1-year suicidal ideation.

## Discussion

### Associations of right rostral temporal thalamus rs-FCN with spatial planning and suicidality

Among the 47 group-level hubs (23 HC hubs, 27 StD hubs, and 26 MDD hubs), the centralities of the right rostral temporal thalamus were lower in MDD than in HC and StD (Fig. 2 and Table 2). This is in accordance with a previous study showing weaker rs-FC of the rostral temporal thalamus with the left lingual gyrus in bipolar disorder (either depressive or remitted status) than in HC^79^. After a body–mind relaxation meditation, both MDD and HC showed decreased FC between the left rostral temporal thalamus and the left inferior occipital cortex^80^. The rostral temporal thalamus, as defined in the Brainnetome Atlas^58^, covers parts of the anterior thalamic complex, which projects to limbic areas in the medial temporal lobe and parts of the dorsomedial thalamic nucleus (related to emotional behavior, memory, attention, and higher cognitive functioning of organization and planning), which receives inputs from the temporal lobe^79^ and projects to the prefrontal cortex and limbic system.

The current study revealed associations of spatial planning (OTSMLC5) with the z-transformed centralities of the right rostral temporal thalamus [r = 0.262 (*P* = 0.020) for DC, r = 0.277 (*P* = 0.014) for EC] (Fig. 3A) and suicidal ideation in StD and MDD within the previous year (Fig. 4A). Impaired executive function in domains such as decision-making, planning, response inhibition, selective attention, working memory, and verbal fluency^54,81^ is a key feature of MDD that has been linked to the risk of suicidal ideation and attempts^82^. First, impaired spatial planning measured using the OTS outcome measure “problems solved on first choice” was associated with a higher risk of suicide attempts within the upcoming year^54^. Second, adolescents with depression and a history of suicidal attempts within the last year showed impaired problem-solving and set-shifting (measured using the Wisconsin Card Sorting Test) and working memory (assessed using the digit span subtest of the Wechsler Adult Intelligence Scale, Fourth Edition) than HC and a history of non-suicidal self-injury^83^. Cognitive inflexibility poses additional difficulties in terms of interpreting and managing life events^84^ and could be a risk factor for suicide in depression^85^.

### Visuospatial strategy with rs-FCN centrality of the right dmPFC

Among all 47 group-level hubs, the centralities of the right dmPFC (BA 6) were higher in the StD group than in both the MDD and HC groups (Fig. 2 and Table 2). Focal brain lesions of the left dmPFC are associated with more severe depressive symptoms^86^. Conditional knockout of lactate dehydrogenase A from the mouse brain reduces L-lactate levels and neuronal excitability in the dmPFC, promoting depressive-like behaviors^87^. Additionally, the depressive symptoms anhedonia, avolition^88–90^, rumination^91^, and apathy^92^ have been linked to dmPFC dysfunction. In one study, hypoactivation of the left dmPFC was related to poorer emotional regulation and an increased likelihood of subsequent mood episode relapse during a 1.5-year follow-up of bipolar disorder compared with HC^93^. Additionally, in adolescents with preschool-onset depression, recognition of fear and surprise during emotional regulation of sadness was accompanied by greater functional activation of the dAI and dmPFC^94^. In another study, rs-FC between the dmPFC and insula was weaker in MDD than in HC^95^. By contrast, increased rs-FC of the dmPFC with the rostral anterior cingulate cortex in response to mindfulness induction was related to emotion regulation^96^. The dmPFC has served as a target of repetitive transcranial magnetic stimulation^97,98^ and intermittent theta burst stimulation^98–100^ for the treatment of anhedonia, avolition, and blunted affect in depression.

The present study revealed a negative association between the SWMBE (visuospatial working memory and strategy) and z-transformed centralities of the right dmPFC (BA 6) [r =  − 0.317 (*P* = 0.004) for DC, r =  − 0.243 (*P* = 0.031) for EC] (Fig. 3B). The dmPFC is the site of convergence for the cognitive control and affect-triggering networks and thereby plays a critical role in both the generation and regulation of emotion^101^. Variance in the rs-FC strength of the dmPFC with other regions, including dorsal and ventral attention networks and the subcortical network, could explain the severity of depressive rumination in both the subclinical population and MDD^35^. Reduced rs-FC strength of the dmPFC with the basolateral amygdala, and of the node strength of the basolateral amygdala, was found in HC after acute stress, but not in MDD^102^. In another study, treatment-resistant depression exhibited significantly reduced oxyhemoglobin activation changes in the dmPFC when performing a verbal fluency task assessed with near-infrared spectroscopy^103^. Further, functional activation of the dmPFC during response inhibition in the SST, which reflects both the severity of depressive symptoms and the resilience of young adults without symptoms, was influenced by the interplay between childhood maltreatment and a family history of alcohol use disorder^104^. In terms of vulnerability to depressive symptoms, a lower level of social trust (which is related to depressive symptoms) was associated with lower gray matter density of the dmPFC in community-dwelling adults^105^.

### Response inhibition and sustained attention: association with rs-FCN centrality of the left dAI

Among all 47 group-level hubs in this study, the centrality of the left dAI in the rs-FCN was higher in the StD group than in both the MDD and HC groups (Fig. 2 and Table 2). This is in line with previous studies that showed associations of the dAI with depressive symptom severity and improvement. First, focal brain lesions in the bilateral AI were associated with more severe depressive symptoms^86^. Second, higher EC of the left dAI in the rs-FCN was associated with more severe depressive symptoms in Parkinson’s disease^106^. Third, depressive symptoms in a diagnosis of either MDD or bipolar disorder were related to inhibitory rs-FC between the AI and the precuneus^107^. Fourth, a significant reduction of depressive symptoms in MDD after sertraline pharmacotherapy was accompanied by decreased rs-FC of the insula with the nucleus accumbens^108^. Fifth, a change in the rs-FC of the left AI with the dorsal anterior cingulate cortex after treatment with repetitive transcranial magnetic stimulation explained the improved emotional health seen in traumatic brain injury^109^.

This study also showed a positive correlation of z-transformed centralities in the left dAI with the SSTSSRT (response inhibition) [r = 0.228 (*P* = 0.044) for DC, r = 0.242 (*P* = 0.031) for EC] (Fig. 3C) and a positive correlation of the z-transformed centralities of the left dAI with the RVPA′ (detection sensitivity in the RVP; sustained attention) [r = 0.245 (*P* = 0.030) for DC, r = 0.263 (*P* = 0.019) for EC] (Fig. 3D). The dAI has functional connections to frontal, anterior cingulate, and parietal areas and is involved in cognitive control processes^110^. Stronger rs-FC of the bilateral insula, supramarginal gyrus, and dorsal anterior cingulate cortex in MDD than in HC was sustained even after the remission of depressive symptoms^111^. Dynamic functional network connectivity analyses have revealed more diverse time-varying FC patterns of the dAI with other brain regions^112^ across multiple task domains^113^. Compared with HC, StD show blunted activity of the bilateral AI when choosing to exert effort to obtain rewards but increased willingness and greater activation of the bilateral AI when choosing to exert effort on the behalf of others^114^.

### Limitations

The current study had some limitations. First, the study used a cross-sectional design. Therefore, the longitudinal trajectories of clinical symptoms^115,116^ and executive functioning in StD must be examined in further studies. Second, the static rs-FCN estimated in the current study cannot be used to explore the possibility of multiple time-varying phases of dynamic rs-FCN in StD. Future studies acquiring brain MRI data with higher temporal resolution^117^ may yield more information. Third, detailed information on how insufficient visuospatial planning contributes to hopelessness and suicidal ideation in StD and MDD was lacking in the current study. Additional studies using projective psychological tests^118,119^ could provide more data.

## Conclusions

Taken together, the results suggest that the suicidality of StD is associated with the executive functions of spatial planning and problem-solving. The executive function of StD is associated with the centralities of the thalamus, dmPFC, and dAI in the rs-FCN. Treatment approaches to improve the executive function, hopelessness, and suicidality of StD must be developed in future studies.

## Data Availability

Participant-level data is not publicly available, but can be accessed by researchers who meet the criteria for access to de-identified sensitive data via request to the corresponding author (jhjang602@naver.com), under the term of clinical research ethics committee of Seoul National University College of Medicine and Hospital.
